# Reply to Ignazio Condello et al

**DOI:** 10.1093/icvts/ivaf202

**Published:** 2025-09-04

**Authors:** Manel Tauron-Ferrer

**Affiliations:** Cardiac Surgery Department, Hospital de la Santa Creu i Sant Pau (HSCSP), IIB Sant Pau, Universitat Autónoma de Barcelona, Barcelona 90 08025, Spain; CIBER-CV, ISCIII, Madrid

Dear Editor,

We thank Dr Condello for his valuable comments on our study regarding how cardioplegia administration routes and de-airing maneuvers could influence myocardial protection.^1^

As Dr Condello noted, our study showed heterogeneity in the route of cardioplegia administration between the Buckberg (BS) and Del Nido (DNS) groups.^2^ Those differences were expected when comparing both solutions. The aim of the study was to compare Buckberg and Del Nido cardioplegia in aortic valve replacement, but the idea was to compare not only the solutions but also the myocardial protection strategies.^3^ Buckberg cardioplegia is usually a multiple dose, antegrade and retrograde combined solution, and Del Nido is usually a single shoot antegrade cardioplegia, using direct ostia cannulation only in those patients with significant aortic regurgitation. Our results and results from other trials comparing both solutions showed that a single-shoot antegrade strategy with Del Nido solution shows no differences compared with the multiple dose, multiple route Buckberg strategy. As pointed in the discussion, we believe that this could simplify the surgical procedure, making it particularly attractive in the minimally invasive setting.

We absolutely agree with our colleagues that shorter CBP and cross-clamp times should be expected using Del Nido solution, as re-dosing interruptions are avoided. Although they have not reached statistical significance, we have found slightly shorter times in Del Nido group (CBP time 78 vs 74 min). As Dr Condello suggests, this might be explained by the use of retrograde cardioplegia to re-dose in the BS group, and we also think that the typically short duration of aortic valve replacement may reduce the need for multiple BS doses, diminishing group differences.

Dr Condello also mentioned perioperative aspects that could influence spontaneous rhythm recovery and ventricular fibrillation rates after cross-clamp removal.^4^ In our study de-airing maneuvers, ventilation strategies, and CBP venting are the same as in our usual clinical practice, no matter which cardioplegia is used. Intraoperative CO_2_ is also routinely used, as it is intraoperative transoesophageal echocardiography to assess correct de-airing, so we believe that those aspects have not influenced our results, as they are equal in both groups. We agree with Dr Condello that those maneuvers could influence spontaneous rhythm recovery and ventricular fibrillation after cross-clamp removal, and further studies comparing different strategies regarding those maneuvers would improve our knowledge on how de-airing impacts myocardial protection. We believe that lidocaine (as a class Ib antiarrhytmic) contained in Del Nido solution may have some impact on ventricular fibrillation after cross-clamp removal. Regarding air embolism and de-airing, we have the impression that it may be also related with cardioplegia route and re-dosing. The need for multiple cardioplegia doses with antegrade and retrograde use could increase the risk of coronary air embolism during the administration of maintenance doses, making de-airing maneuvers even more important when using multiple dose cardioplegic strategies. That could make the single dose strategy even more attractive, as it could simplify not only the surgical procedure but also de-airing maneuvers, minimizing the myocardial injury related with air embolism.
